# Attachment Avoidance Mediates the Relationship Between Relatedness Frustration and Social Networking Sites Addiction: Conscientiousness and Neuroticism as Moderators

**DOI:** 10.3390/bs14111068

**Published:** 2024-11-08

**Authors:** Shu Zhong, Ri Hai, Nigela Ahemaitijiang, Xinyue Wang, Yunxiang Chen, Xiangping Liu

**Affiliations:** 1 Center for Counseling and Psychological Development, Tsinghua University, Beijing 100089, China; nigela@tsinghua.edu.cn; 2 Department of Applied Social Sciences, The Hong Kong Polytechnic University, Hong Kong, China; 21055122g@connect.polyu.hk; 3 Department of Psychological and Cognitive Science, Tsinghua University, Beijing 100086, China; wxy21@mails.tsinghua.edu.cn; 4 School of Psychology, Beijing Normal University, Beijing 100875, China; chen1033@mail.bnu.edu.cn (Y.C.); lxp599@163.com (X.L.)

**Keywords:** social networking sites addiction, relatedness frustration, attachment avoidance, big five personality

## Abstract

Social Networking Sites Addiction (SNSA) has become increasingly prevalent among university students, posing significant challenges to their mental health and academic performance. The psychological mechanisms and personality traits contributing to this addiction are not yet fully understood. This study explored the mediating effect of attachment avoidance (AA) between relatedness frustration (RF) and SNSA, as well as the moderating roles of Conscientiousness and Neuroticism from the Big Five personality traits. Data were collected from 489 university students (M = 18.81, SD = 0.92) using the Relatedness Frustration Scale, Ten-Item Personality Inventory in Chinese, Experiences in Close Relationships Scale (ECR) Short Form, and Social Networking Sites Addiction Scale. The findings were as follows: (1) RF significantly predicted SNSA; (2) RF significantly predicted AA, which in turn significantly predicted SNSA, indicating a partial mediation; and (3) Conscientiousness and Neuroticism moderated the effect of RF on SNSA, with higher levels of these traits amplifying the positive effect of RF on SNSA. The study concluded that individuals facing RF may avoid real-life attachments and seek connections online, increasing the risk of SNSA, especially for those with high levels of Conscientiousness and Neuroticism.

## 1. Introduction

Social networking sites (SNSs) refer to web-based services where individuals, organizations, and their relationships form different social networking structures [1]. Though the nature of these platforms may vary from site to site, they generally satisfy individuals’ affiliation needs, promote interpersonal communication, and help form a positive self-concept [2]. However, excessive use of SNSs can lead to Social Networking Sites Addiction (SNSA), where individuals become overly invested in SNS activities, neglect real-life interpersonal relationships, and experience negative psychological and social consequences [3,4]. SNSA is a behavioral addiction characterized by compulsive use of social networks despite negative impacts on daily life and functioning, such as impaired psychological well-being, academic performance, and social functioning [5].

SNSA is one type of Internet-related addiction, which falls under the broader category of behavioral addictions. Other types of internet-related addictions include Internet Gaming Disorder (IGD), smartphone addiction, and Online Gambling Addiction. Each form of addiction has its own specific triggers and manifestations. For example, Internet Gaming Disorder is more often associated with escapism and the pursuit of achievement, while smartphone addiction is linked to productivity tools and the need for instant gratification [6,7]. In contrast, SNSA primarily involves a dependency on virtual social interactions to compensate for deficiencies in real-life relationships, further exacerbating loneliness and social anxiety [3].

This issue is particularly prevalent among Gen Z university students, a cohort that has grown up with digital technology and social media. Due to their reliance on smartphones and social networking sites, many young people are at risk of developing addictive behaviors, which has drawn significant research attention [5].

Attachment styles are associated with SNSA. For example, peer attachment negatively predicts SNSA, with mediators such as social anxiety and fear of negative evaluation playing key roles [8,9]. Attachment styles can be divided into attachment anxiety (fear of rejection and abandonment) and attachment avoidance (discomfort with intimacy and dependence) [10,11]. Both of these styles have been found to mediate the relationship between childhood trauma and problematic SNS usage [12]. For instance, individuals with high attachment anxiety are driven to seek social connections on SNSs, leading to excessive usage [13,14,15]. However, the relationship between attachment avoidance and SNSA is less clear. Some research suggests that individuals with high levels of attachment avoidance prefer to reduce interpersonal connections and minimize online social interactions [16,17,18], while others also point out that online gaming spaces provide secure contexts for the attachment avoidance [19], creating a contradiction in the literature.

Another critical factor related to SNSA is relatedness frustration. According to the Basic Psychological Needs Theory, humans share three universal basic psychological needs: autonomy, relatedness, and competence [20]. Frustration of these needs leads to maladjustment and psychological issues, including internet addiction [21]. For instance, frustrated relatedness needs to predict high SNSA, as individuals may turn to online social networks to compensate for unmet social connections in real life [22]. Relatedness frustration also predicts other forms of problematic internet use [23], such as Internet Gaming Disorder [6,23]. We propose that attachment avoidance mediates the relationship between relatedness frustration and SNSA, suggesting that individuals who avoid real-life connections due to negative relationship setbacks may develop excessive SNS use to fulfill their relational needs online. This mediating mechanism forms a key part of the current study’s hypothesis.

The Big Five personality traits also influence the degree of internet-related addictions, including SNSA. While most existing research focuses on their main effects, our study highlights their moderating role in the relationship between relatedness frustration and SNSA. However, the direction of influence varies across different studies. For instance, Neuroticism and Extraversion are often identified as positive predictors of internet addiction and related behaviors [24,25,26], while Openness is typically associated with curiosity and tends to show positive correlations with internet addiction [7,26]. Agreeableness plays a more complex role, negatively predicting online game addiction [24,27] but positively predicting smartphone addiction [7]. Conscientiousness, meanwhile, has shown mixed results, with some studies associating it with lower addiction risk due to better time management and self-discipline [24,28], while others indicate that, under stress, conscientious individuals may engage in addictive behaviors to cope with failing to meet their high standards [29]. The relationship between Conscientiousness and addiction may vary by addiction type, as it negatively predicts gaming addiction but may complicate smartphone addiction, especially when individuals use their devices for productivity [7]. More importantly, the interaction between Conscientiousness and Neuroticism can exacerbate addictive tendencies, particularly when stress or perfectionism is involved [30,31,32]. Given these mixed findings, it is essential to consider the context and personality interactions when studying addiction. Conscientiousness and Neuroticism, in particular, have demonstrated significant roles in emotional regulation and self-control, especially in stressful contexts [33]. Research suggests that these two traits often interact to shape individuals’ behavioral responses to stress, with high Conscientiousness potentially leading to greater pressure due to perfectionism and high Neuroticism exacerbating emotional instability [34]. This interaction helps explain their unique role in the relationship between relatedness frustration and SNSA, while other Big Five traits like Openness, Agreeableness, and Extraversion are less directly implicated in moderating stress-induced behaviors [33,34].

Thus, compared to exploring the direct relationship between personality and SNSA, it is more crucial to explore the moderating role of personality traits, especially Conscientiousness and Neuroticism, from a multi-factorial perspective. Our research proposes that attachment avoidance mediates the relationship between relatedness frustration and SNSA, and Neuroticism and Conscientiousness moderate the pathway from attachment avoidance to SNSA.

**Hypothesis** **1 (H1).** *Attachment avoidance mediates the relationship between relatedness frustration and SNSA.*

**Hypothesis** **2 (H2).** *High levels of Neuroticism and Conscientiousness strengthen the mediation pathway between attachment avoidance and SNSA.*

This research addresses a significant gap in understanding the relationship between psychological stressors and SNSA by examining how relatedness frustration directly influences SNSA. While much of the existing literature has focused on general predictors of internet addiction, few studies have explored the specific role of relational stress in contributing to addictive behaviors. By identifying attachment avoidance as a key mediator in this relationship, this research offers deeper insights into the psychological mechanisms that link interpersonal challenges to problematic social network use.

Moreover, our study contributes to the field by investigating how personality traits, specifically Conscientiousness and Neuroticism, moderate the effect of relatedness frustration on SNSA. This adds a critical layer of complexity to existing models of internet addiction, revealing how individual differences in emotional regulation and self-control shape addiction risk in high-stress environments.

Finally, these findings have important implications for the development of targeted interventions. By recognizing the moderating role of personality traits, we suggest that future interventions should focus on improving emotional regulation strategies, especially for individuals prone to high Conscientiousness and Neuroticism, to better manage relational stress and reduce the risk of SNSA.

## 2. Method

### 2.1. Participants

A total of 489 Chinese university students (299 females) aged 17–28 (M = 18.81, SD = 0.92) were recruited by online questionnaire in the current study; the age mainly fell between 18 and 20 years (18, *n* = 173; 19, *n* = 227; 20, *n* = 67). The educational background of participants’ parents (rating from “junior high and below” = 1 to “doctorate” = 5) was also collected; Father’s Education Background (FEB) mainly fell in “undergraduate/ junior college” (*n* = 210), then followed by “junior high and below” (*n* = 135); Mother’s Education Background (MEB) mainly fell in “junior high and below” (*n* = 164), then followed by “undergraduate/ junior college” (*n* = 162). Parental Average Education Background (PAEB) was then calculated (M = 2.18, SD = 0.89).

### 2.2. Measurement

#### 2.2.1. Relatedness Frustration Scale

The Relatedness Frustration Scale (RFS) from the Need Frustration and Need Satisfaction Scales (NFNSS) [35] was adopted to assess the degree to which individuals feel isolated, unsupported, or rejected in their relationships (e.g., *I feel excluded from the group I want to belong to*). Participants rate 4 items on a 5-point Likert scale ranging from 1 (*completely untrue*) to 5 (*completely true*). The average score of 4 items represents the participant’s level of frustration in relationships. In the current study, the Cronbach’s α for the RFS = 0.74. It has also been validated in multiple cultural contexts to ensure their reliability and generalizability [35].

#### 2.2.2. The Ten-Item Personality Inventory in Chinese

Lu et al. amended the Chinese version of the Ten-Item Personality Inventory to investigate the Big Five personality traits of the Chinese [36,37]. Every 2 items (including 1 reverse scoring item) measure one personality trait (i.e., Openness, Conscientiousness, Extraversion, Agreeableness, and Neuroticism), hence a total of 5 items (items 2, 4, 6, 8, and 10) need to be reversely scored before data analysis. Each item lists two words that positively or negatively represent the measured dimension (for example, “*Extraverted, enthusiastic*” and “*Reserved, quiet*” are the two items of Extraversion), and participants will rate between 1 (*completely untrue*) and 5 (*completely true*). The total score of each dimension after final scoring indicates the individual’s characteristic on this trait. Each dimension of the Ten-Item Personality Inventory has relatively low internal consistencies (Cronbach’s alphas = 0.45, 0.50, 0.68, 0.40, and 0.73, for Openness, Conscientiousness, Extraversion, Agreeableness, and Neuroticism), but the test–retest showed substantial reliability correlations (mean *r* = 0.72, six-week), good convergent correlations with the corresponding dimensions in the original Big-Five Inventory were also found (*r* = 0.65, *p* < 0.01, for Openness; *r* = 0.75, *p* < 0.01, for Conscientiousness; *r* = 0.87, *p* < 0.01, for Extraversion; *r* = 0.70, *p* < 0.01, for Agreeableness; and *r* = 0.81, *p* < 0.01, for Neuroticism), making it a good measure of individual personality traits [37].

#### 2.2.3. The Experiences in Close Relationship Scale (ECR) Short Form

The avoidance subscale of Experiences in Close Relationships—Short Form (ECR-S) was adopted to measure participants’ attachment styles. Deriving from the Chinese version of the Experiences in Close Relationship Scale (ECR) [11,38], the ECR-S is a 12-item measure designed to assess individual differences from two aspects: attachment anxiety (6 items) and attachment avoidance (6 items). The Cronbach’s *α* for the anxiety subscale and for the avoidance subscale are 0.77 and 0.78, respectively [11]. A 7-point Likert-type scale ranging from 1 (*disagree strongly*) to 7 (*agree strongly*) is used to rate how well each statement describes people’s typical feelings in close relationships (e.g., *I want to get close to them, but I keep pulling back*). Average scores will be calculated as the index of avoidance. A higher score indicates a higher level of individual avoidance.

#### 2.2.4. Social Networking Sites Addiction Scale

Social Networking Sites Addiction was assessed by the Short-Version Social Networking Websites Addiction Scale (SNWAS) [5]. Chen and colleagues then amended and translated the scale into the Chinese version [13]. The current study has five items with a Cronbach’s α = 0.81. Participants were required to evaluate from 1 (*strongly disagree*) to 7 (*strongly agree*) on the items (e.g., *When I am not using social networking websites, I often feel agitated*), and an average score was calculated as the level of Social Networking Sites Addiction.

## 3. Results

### 3.1. Descriptive Statistics and Correlation Analysis

The independent sample *t*-test found that the relatedness frustration of male university students (M = 2.67, SD = 0.75) was significantly higher than that of female university students (M = 2.34, SD = 0.74; *t* = 4.76, *p* < 0.001, Cohen’s d = 0.44). There were no significant gender differences in FEB, MEB, PAEB, attachment avoidance, or Social Networking Sites Addiction. The mean, standard deviation, and correlation analysis of the main variables are shown in Table 1.

Results indicated that (1) parents’ educational background (FEB, MEB, and PAEB) was found to be correlated with multiple variables, so its influence as a covariate will be eliminated in further analysis; (2) significant correlations between relatedness frustration (RF), attachment avoidance (AA), and Social Networking Sites Addiction (SNSA) were found (*r* = 0.21, *p* < 0.01, between RF and AA; *r* = 0.33, *p* < 0.01, between RF and SNSA; *r* = 0.21, *p* < 0.01, between SNSA and AA); (3) when considering the Big Five traits, Neuroticism was correlated with relatedness frustration (*r* = −0.26, *p* < 0.01), attachment avoidance (*r* = −0.15, *p* < 0.01), and SNSA (*r* = −0.11, *p* < 0.05).

Though there was no significant correlation between Conscientiousness and the three experimental variables above, given that it is correlated with online gaming addiction and smart phone addiction, it will also be included in further analysis.

### 3.2. Tests of Moderated Mediating Effect

Model 7 from the PROCESS plug-in developed by Hayes was adopted to explore the mediating and regulating effects of variables [39]. PROCESS is an add-on to the SPSS 27.0 software that simplifies the analysis of conditional process models. PAEB was controlled as a covariate to eliminate its influence on other variables. A total of 2 structures were eventually found to be significant in all pathways:

(1) RF as the predictor, AA as the mediator, SNSA as the criterion, and Conscientiousness as the regulator, named Structure 1, moderated mediating effects see Table 2.

(2) RF as the predictor, AA as the mediator, SNSA as the criterion, and Neuroticism as the regulator, named Structure 2, moderated mediating effects see Table 3.

Results showed that in Structure 1: (1) the direct relationship between RF and SNSA reached a significant level (β = 2.22, *p* < 0.01, 95%CI = [1.58 2.86]); (2) AA significantly predicted SNSA (β = 0.13, *p* < 0.01, 95% CI = 0.05–0.21); (3) the interaction effect of RF and Conscientiousness was found to be significant and positive (β = 0.72, *p* < 0.05); the simple slope test reflects that the indirect impact of RF on SNSA strengthens when Conscientiousness rises to a medium or high level (see Table 4).

In Structure 2: (1) the direct effect between RF and SNSA reached the same significant level as in Structure 1 (β = 2.22, *p* < 0.01, 95% CI = 1.58–2.86); (2) RF significantly predicted AA (β = 6.01, *p* < 0.01), AA significantly predicted SNSA (β = 0.13, *p* < 0.01, 95% CI = 0.05–0.21); (3) the interaction effect of RF and Neuroticism was found to be significant and negative (β = −0.85, *p* < 0.01). The simple slope test reflects that the indirect impact of RF on SNSA strengthens when Conscientiousness rise to a medium or high level (see Table 4).

**Figure 1:**
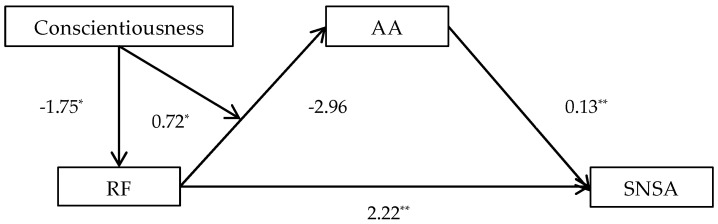
Moderated mediating effects of Structure 1. * *p* < 0.05, ** *p* < 0.01.

**Figure 2:**
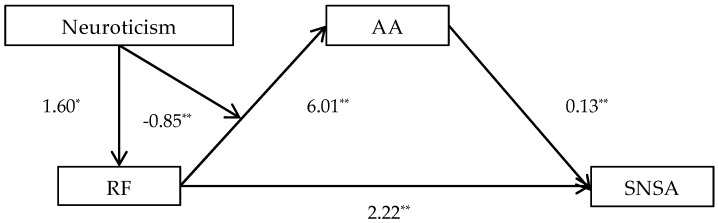
Moderated mediating effects of Structure 2. * *p* < 0.05, ** *p* < 0.01.

The moderated mediating effects of Structure 1 and Structure 2 were separately shown in Figure 1 and Figure 2.

## 4. Discussion

This study examined the relationship between relatedness frustration and Social Networking Sites Addiction (SNSA), with a particular focus on the mediating role of attachment avoidance and the moderating effects of the Big Five personality traits, especially Conscientiousness and Neuroticism. Our findings indicated that relatedness frustration significantly predicts SNSA, which aligns with previous findings and is consistent with Basic Psychological Needs Theory [6,13,21,22,23]. Attachment avoidance partially mediated this relationship, suggesting that individuals experiencing relational frustrations may avoid real-life attachments and seek psychological substitutes online, increasing their SNSA risk. This supports previous research while clarifying the role of attachment avoidance, challenging earlier claims that it has no relationship with SNSA [16,17,18]. The anonymity and lack of deep interpersonal interaction in online settings make SNS an appealing alternative, allowing individuals to avoid negative evaluations and other discomforts of face-to-face communication [9,40,41]. Consequently, spending excessive time online to compensate for real-world relational losses may make individuals more vulnerable to SNSA.

### 4.1. Mediating and Moderating Mechanisms

The study further demonstrated that Conscientiousness and Neuroticism moderated the effects of relatedness frustration on SNSA. This finding provides a more nuanced insight into how personality traits influence the relationship between psychological need frustration and addictive behaviors. High levels of Conscientiousness appear to exacerbate the effects of relatedness frustration on SNSA, contrary to the typical association of Conscientiousness with self-discipline and lower addiction risk [24,28]. This is likely due to the perfectionism and heightened self-expectations associated with Conscientiousness, which may lead individuals to feel more intensely frustrated in their relationships when they fail to meet their high standards [29,42]. Under such circumstances, conscientious individuals might turn to SNSs as a way to cope with this frustration, thus contributing to the paradoxical finding that Conscientiousness can, in some cases, increase the risk of addiction. We found the path from relatedness frustration to attachment avoidance is not significant. Considering that the two factors have a positive correlation, such non-significance may be influenced by the moderating effect of Conscientiousness.

Similarly, Neuroticism also exacerbated the impact of relatedness frustration on SNSA, which is consistent with previous research showing that neurotic individuals tend to be more sensitive to social rejection and frustration, leading to maladaptive coping strategies, such as excessive SNS use [25]. It is noteworthy that medium and high levels of Neuroticism maintained the same moderating effect in the simple slope test; such a phenomenon reinforces the idea that Neuroticism is a key risky factor in the formation of SNSA. Reducing neurotic tendencies may be a crucial intervention point for preventing SNSA among individuals with high relational frustrations.

### 4.2. Theoretical and Practical Implications

These findings have significant implications for both theory and practice. Theoretically, this study contributes to a deeper understanding of the psychological mechanisms underlying Social Networking Sites Addiction (SNSA) by highlighting the crucial role of relatedness frustration as a key antecedent. While existing research often emphasizes broad predictors of internet addiction, this study addresses the specific impact of relational stress, thereby filling a gap in the literature on addiction behaviors linked to interpersonal dynamics. Additionally, the identification of attachment avoidance as a mediator offers valuable insights into how emotional avoidance strategies drive individuals toward excessive social network use, particularly when faced with relational challenges.

Moreover, the study underscores the importance of considering personality traits not only as direct predictors but as moderators in the relationship between psychological needs and addictive behaviors. By examining Conscientiousness and Neuroticism as moderating factors, this research provides a more nuanced framework for understanding how individual differences in emotional regulation and self-control influence susceptibility to addiction. This multi-factor approach expands current theoretical models and suggests that personality traits can either mitigate or exacerbate the effects of relational stress on addiction, depending on individual emotional tendencies.

Practically, these insights inform the development of targeted interventions aimed at reducing SNSA. For individuals with high Conscientiousness, interventions should focus on addressing perfectionistic tendencies that heighten relational frustration, offering alternative coping mechanisms that do not involve social network use. Similarly, for those with high Neuroticism, interventions should prioritize emotion regulation strategies that help individuals manage relational frustrations without resorting to compulsive social media engagement. Specifically, strategies that promote secure attachment styles and address relatedness frustration may be effective in mitigating addiction risks. By enhancing emotional coping skills and reducing dependence on social networks as an escape, such interventions can significantly lower the likelihood of problematic SNS use.

### 4.3. Limitations and Future Research

Despite its contributions, this study has several limitations. First, the cross-sectional design limits our ability to infer causality between relatedness frustration, attachment avoidance, and SNSA. Longitudinal studies are needed to establish the temporal order of these relationships and confirm the mediating and moderating effects over time. Second, the reliance on self-report measures may introduce bias, as participants might have provided socially desirable responses. Future research should incorporate multi-method approaches, including behavioral assessments, to validate these findings and mitigate potential bias. Additionally, future research should explore other potential moderators, such as additional personality traits or social factors, which may interact with relatedness frustration and SNSA. Third, our conclusions may not fully address the broad spectrum of online addiction behaviors. Different types of online addictions, such as gaming addiction, internet gambling, and smartphone addiction, may have distinct mechanisms, requiring more detailed exploration in future studies. Addressing these various types of online addictions will provide a more comprehensive understanding of the broader issue and allow for more specific intervention strategies. Finally, experimental research manipulating relatedness frustration and examining its direct effects on SNS use could offer valuable insights into the causal mechanisms driving SNSA.

## 5. Conclusions

In conclusion, this study highlights the significant role of relatedness frustration in predicting SNSA, with attachment avoidance acting as a partial mediator and personality traits such as Conscientiousness and Neuroticism moderating this relationship. These findings contribute to a deeper understanding of the psychological factors underlying SNSA and suggest several pathways for intervention, particularly in addressing the relational and personality-driven aspects of addiction.

## Figures and Tables

**Table 1:** Correlation analysis of main variables.

	M	SD	1	2	3	4	5	6	7	8	9	10	11
1 FEB	2.28	1.01	-										
2 MEB	2.08	0.95	0.67 **	-									
3 PAEB	2.18	0.89	0.92 **	0.91 **	-								
4 Relatedness Frustration	2.47	0.76	−0.08	−0.08	−0.09 *	-							
5 Extraversion	6.29	0.87	0.04	0.10 *	0.07	0.04	-						
6 Agreeableness	7.27	1.32	0.07	0.07	0.08	−0.05	0.26 **	-					
7 Conscientiousness	6.21	1.14	−0.08	0.01	−0.04	0.06	0.21 **	0.23 **	-				
8 Openness	6.57	1.00	−0.12 **	−0.05	−0.09 *	−0.01	0.20 **	0.13 **	0.15 **	-			
9 Neuroticism	5.54	1.09	0.02	0.03	0.03	−0.26 **	−0.19 **	−0.12 **	−0.12 **	−0.27 **	-		
10 Attachment Avoidance	24.33	5.96	−0.09	−0.09 *	−0.10 *	0.21 **	−0.04	−0.02	0.03	0.05	−0.15 **	-	
11 SNSA	19.86	5.74	−0.13 **	−0.15 **	−0.15 **	0.33 **	0.04	0.04	−0.00	0.09	−0.11 *	0.21 **	-

Note: * *p* < 0.05, ** *p* < 0.01.

**Table 2:** Moderated mediating effects of Structure 1.

Mediator Variable Model	Coefficient	SE	*t*	LLCI	ULCI
Constant	32.50				
RF → AA	−2.96	1.88	−1.58	−6.64	0.73
Conscientiousness → AA	−1.75 *	0.77	−2.26	−3.26	−0.23
RF × Conscientiousness → AA	0.72 *	0.30	2.45	0.14	1.30
**Dependent variable model**	**Coefficient**	**SE**	***t***	**LLCI**	**ULCI**
Constant	12.87				
RF → SNSA	2.22 **	0.33	6.81	1.58	2.86
AA → SNSA	0.13 **	0.04	3.09	0.05	0.21

Note: * *p* < 0.05, ** *p* < 0.01.

**Table 3:** Moderated mediating effects of Structure 2.

Mediator Variable Model	Coefficient	SE	*t*	LLCI	ULCI
Constant	13.19				
RF → AA	6.01 **	1.41	4.27	3.24	8.77
Neuroticism → AA	1.60 *	0.68	2.35	0.26	2.93
RF × Neuroticism → AA	−0.85 *	0.25	−3.43	−1.34	−0.36
**Dependent variable model**	**Coefficient**	**SE**	***t***	**LLCI**	**ULCI**
Constant	12.87				
RF → SNSA	2.22 **	0.33	6.81	1.58	2.86
AA → SNSA	0.13 **	0.04	3.09	0.05	0.21

Note: * *p* < 0.05, ** *p* < 0.01.

**Table 4:** Conditional indirect effects of Conscientiousness and Neuroticism.

Conditional Indirect Effects of Conscientiousness	Effect	SE	LLCI	ULCI
Conscientiousness Low Mean − 1 SD	0.08	0.08	−0.05	0.26
Conscientiousness Mean	0.18	0.09	0.04	0.37
Conscientiousness High Mean + 1 SD	0.29	0.12	0.07	0.54
Index of Moderated Mediation	0.09	0.06	0.01	0.23
**Conditional Indirect Effects of Neuroticism**	**Effect**	**SE**	**LLCI**	**ULCI**
Neuroticism Low Mean − 1 SD	0.22	0.11	0.05	0.47
Neuroticism Mean	0.11	0.08	0.01	0.31
Neuroticism High Mean + 1 SD	0.11	0.08	0.01	0.31
Index of Moderated Mediation	−0.11	0.06	−0.23	−0.01

## Data Availability

This study was not preregistered. All data and code have been made publicly available and can be accessed at https://osf.io/grfh7/?view_only=33644a88d6464d84ab7b0816d0cdda24 (accessed on 10 July 2024).
